# Inflammatory–Thrombotic Phenotypes and Outcomes in COVID-19-Associated Pulmonary Embolism: A Single-Center Cohort Study

**DOI:** 10.3390/biomedicines13123064

**Published:** 2025-12-12

**Authors:** Aneta-Rada Dobrin, Voichita Elena Lazureanu, Cristian Oancea, Livia Stanga, Alexandra Herlo, Silvia Alda, Diana Utu, Lucian Flavius Herlo, Monica Licker

**Affiliations:** 1 Doctoral School, “Victor Babes” University of Medicine and Pharmacy, 300041 Timisoara, Romania; aneta.goia@umft.ro (A.-R.D.); flavius.herlo@umft.ro (L.F.H.); 2 Department XIII, Discipline of Infectious Diseases, “Victor Babes” University of Medicine and Pharmacy, 300041 Timisoara, Romania; lazureanu.voichita@umft.ro; 3 Department XIII, Discipline of Pulmonology, “Victor Babes” University of Medicine and Pharmacy, 300041 Timisoara, Romania; oancea@umft.ro; 4 Center for Research and Innovation in Precision Medicine of Respiratory Diseases (CRIPMRD), “Victor Babes” University of Medicine and Pharmacy, 300041 Timisoara, Romania; 5 Discipline of Microbiology, Multidisciplinary Research Center of Antimicrobial Resistance, “Victor Babes” University of Medicine and Pharmacy, 300041 Timisoara, Romania; licker.monica@umft.ro; 6 Faculty of Medicine, “Victor Babes” University of Medicine and Pharmacy, 300041 Timisoara, Romania; silvia.alda@student.umft.ro; 7 Department of Pharmacology, Physiology and Physiopathology, “Victor Babes” University of Medicine and Pharmacy, 300041 Timisoara, Romania; diana.utu@umft.ro; 8 Microbiology Laboratory, “Pius Brinzeu” Emergency Clinical County University Hospital, 300723 Timisoara, Romania

**Keywords:** pulmonary embolism, COVID-19, biomarkers, inflammation, principal component analysis

## Abstract

**Background and Objectives**: Pulmonary embolism (PE) complicating COVID-19 combines viral pneumonia with immunothrombosis, but the relative prognostic value of comorbidities, embolic burden, lung involvement, and inflammatory markers is uncertain. We aimed to characterize clinical profiles and identify independent predictors of respiratory failure and in-hospital mortality in COVID-19–associated PE. **Materials and Methods**: We performed an observational single-center study including 161 adults with RT-PCR–confirmed COVID-19 and CTPA-confirmed acute PE. Demographic data, comorbidities, CT lung involvement, biomarkers, and treatments were compared across outcomes, and multivariable logistic regression and principal component analysis (PCA) were used to model risk. **Results**: In-hospital mortality was 24.8% (40/161), and invasive mechanical ventilation was 18.6% (30/161). Classical PE variables (Wells score, PESI, thrombus location, D-dimer) and comorbidities showed limited discrimination, whereas severe CT lung involvement independently predicted death (OR 4.46; 95% CI 1.55–12.86) and intubation (OR 8.88; 95% CI 2.92–27.04). Each 50 mg/L increase in CRP increased mortality odds by 41% (OR 1.41; 95% CI 1.02–1.96), and a 10-fold rise in IL-6 (log10 IL-6) was associated with six-fold higher mortality (OR 6.10; 95% CI 1.84–20.18). PCA identified a hyperinflammatory–thrombotic component (PC1); per 1-SD increase in standardized PC1, the odds of intubation or death rose 3.5-fold (OR 3.50; 95% CI 2.05–5.99), and event rates across PC1 tertiles were 7.4%, 20.8%, and 57.4%. **Conclusions**: In COVID-19–related PE, integrated immunothrombotic activation and extent of lung involvement, rather than embolic burden alone, drive early critical outcomes and may support refined risk stratification.

## 1. Introduction

Pulmonary embolism (PE) emerged early in the pandemic as a prominent complication of COVID-19, reflecting the profound coagulopathy and endothelial injury associated with SARS-CoV-2 infection. Large multicenter cohorts and meta-analyses have reported PE in roughly 3–8% of hospitalized patients and up to 15–25% of those in intensive care, despite standard thromboprophylaxis [1,2,3]. Recent findings comparing COVID-19–related PE with non-COVID PE, COVID-19 cases showed more extensive parenchymal lung involvement but similar embolic burden and short-term mortality, underscoring the interaction between viral pneumonia and thromboembolic disease [4].

Beyond the acute phase, population-based data indicate that the risk of venous thromboembolism (VTE), including PE, remains elevated for several months after COVID-19, particularly in those with severe disease [5]. This complex epidemiology makes it important to characterize how clinical severity, thrombus distribution, and systemic inflammation jointly shape the course of COVID-19–related PE.

Beyond classical embolic mechanisms, pulmonary thrombosis in situ and microvascular occlusion have been described, blurring the boundary between “typical” PE and COVID-associated pulmonary thrombosis. Autopsy and imaging studies document widespread pulmonary microthrombi, thromboinflammatory lesions, and segmental or subsegmental thrombi that may arise on a background of severe endothelial dysfunction and immunothrombosis [3,6,7,8].

In COVID-19, activation of neutrophil extracellular traps, platelet hyperreactivity, and complement-mediated endothelial injury appear to converge on the pulmonary microvasculature, promoting both macro- and microvascular obstruction [6,7,8]. This “pulmonary vascular phenotype” is reflected in CT pulmonary angiography (CTPA) series, where PE is more often peripheral and multifocal than in non-COVID settings and may coexist with diffuse ground-glass opacities and consolidations [3,9,10]. Such features challenge the traditional dichotomy between hemodynamically significant emboli and “incidental” small-vessel thrombi and complicate risk stratification.

Clinicians managing COVID-19–associated PE must simultaneously contend with acute respiratory failure from viral pneumonia and the hemodynamic and gas-exchange consequences of embolic obstruction. The prognostic value of traditional PE risk tools—such as the Wells score for pre-test probability and PESI/sPESI for mortality risk—may be altered by the background of viral sepsis, extensive parenchymal damage, and profound inflammatory activation. In several COVID-19 cohorts, PE has frequently been diagnosed in patients with low or intermediate clinical suspicion, but markedly elevated D-dimer, and CTPA often reveals predominantly segmental or subsegmental thrombi [1,3,9,10]. At the same time, meta-analytic data show that D-dimer levels are substantially higher in COVID-19 patients with PE than in those without, yet the optimal diagnostic threshold is far higher and more variable than in non-COVID populations, limiting the negative predictive value of conventional cut-offs [10,11,12]. These observations raise questions about how best to integrate pre-test probability scores, D-dimer, and imaging in this specific clinical context.

Inflammatory and coagulation biomarkers, including D-dimer, C-reactive protein (CRP), ferritin, interleukin-6 (IL-6), and procalcitonin, are routinely measured in moderate–severe COVID-19 and frequently inform clinical decision-making. In a large retrospective study of almost 700 hospitalized COVID-19 patients, D-dimer showed only moderate discriminative ability for PE (AUC ≈ 0.77), with extremely high positivity rates at thresholds traditionally used to exclude embolism [11].

Meta-analyses similarly indicate that higher D-dimer levels are consistently associated with PE, but that raising the threshold to improve specificity risks missing clinically relevant events [10,12]. Other studies have emphasized that selecting D-dimer cut-offs based purely on “optimal” Youden indices is clinically unsafe in COVID-19 and that calibration to acceptable failure rates is essential [12]. Against this background, more recent work has shifted toward combining D-dimer with other inflammatory indices (such as neutrophil–lymphocyte ratios, CRP, or composite biomarker scores) and clinical variables to better delineate which patients warrant urgent CTPA and intensified monitoring [13,14,15].

COVID-19 commonly affects individuals with multiple chronic conditions, such as obesity, diabetes, cardiovascular disease, and cancer. These comorbidities not only predispose to severe COVID-19 and VTE but may also influence tolerance to hypoxia and anticoagulation, as well as susceptibility to sepsis and multi-organ failure. In large observational datasets, patients with COVID-19 and PE tend to be older, more often male, and more frequently affected by cardiometabolic disease than COVID-19 patients without PE, yet comorbidity profiles only partially explain the excess risk of respiratory deterioration and death [1,2]. Whether, in patients who already have PE, baseline comorbidity burden meaningfully modifies short-term outcomes beyond the acute inflammatory and respiratory picture remains uncertain, particularly in smaller single-center cohorts. Clarifying this could help clinicians prioritize which information—chronic risk factors versus dynamic physiological and biomarker changes—is most actionable at the bedside.

Therapeutic strategies in COVID-19 have also evolved, with widespread use of corticosteroids, IL-6 inhibitors, IL-1 blockers, and antiviral agents, all of which may affect inflammation, thrombosis, and secondary infection risk. In a large US national cohort, acute PE in hospitalized COVID-19 patients was independently associated with higher in-hospital mortality and greater need for mechanical ventilation, even after adjustment for demographics and comorbidities, and admission D-dimer levels were used to construct a simple prediction calculator [13].

Other studies for PE prediction scores in hospitalized COVID-19 patients highlight only modest performance of repurposed tools such as Wells, Geneva, and Padua scores, and suggest that models incorporating COVID-specific inflammatory and coagulation markers may perform better [14]. In this direction, newer indices such as the C-reactive protein-to-lymphocyte ratio (CLR) have been proposed, with one single-center study showing that elevated CLR, together with higher CRP and D-dimer, strongly predicted PE and mortality in COVID-19 [15]. Instead of trying to infer causal treatment effects from such observational data, they can be used descriptively to understand how advanced therapies cluster with high-risk biomarker patterns and clinical trajectories.

Against this background, we analyzed a single-center cohort of hospitalized COVID-19 patients with imaging-confirmed PE to address three main objectives: (i) to describe the demographic and comorbidity profile of this population; (ii) to compare thrombus location, lung involvement, inflammatory and coagulation biomarkers, and treatments across key clinical outcomes (invasive ventilation and in-hospital death); and (iii) to explore independent associations between these variables and adverse outcomes using multivariable models.

## 2. Materials and Methods

### 2.1. Study Design and Population

We conducted an observational, single-center cohort study using an anonymized dataset of consecutive adults hospitalized with COVID-19 and objectively confirmed PE. All patients had SARS-CoV-2 infection and underwent imaging—typically CT pulmonary angiography—demonstrating acute pulmonary thromboembolism. The dataset included 161 patients and was originally assembled from routine clinical records at a tertiary hospital; for this educational project, all identifiers were removed before analysis.

The analytic sample included all 161 patients with complete information on sex, age, thrombus location, pulmonary involvement score, key laboratory variables (D-dimer, CRP, ferritin, IL-6), and in-hospital outcomes (intubation and death). No additional exclusion criteria were applied in order to reflect real-world heterogeneity in disease severity, comorbidities, and treatments.

### 2.2. Clinical Variables

Demographic characteristics (age, sex, and place of residence) and baseline comorbidities were extracted from the admission records and discharge summaries. Comorbidities of interest included obesity (body mass index ≥ 30 kg/m^2^ or documented diagnosis), diabetes mellitus, active cancer (solid or hematologic malignancy under active treatment or with metastatic disease), chronic heart failure (New York Heart Association class ≥ II), chronic pulmonary hypertension (echocardiographic or hemodynamic diagnosis in the source chart), and prior chronic anticoagulation (vitamin K antagonists, direct oral anticoagulants, or long-term low-molecular-weight heparin). Vaccination status against COVID-19 (unvaccinated vs. ≥1 vaccine dose prior to admission) was also collected.

The timing of thromboembolic events was characterized by the number of days from COVID-19 symptom onset to PE diagnosis, defined as the date of the first CTPA study confirming acute PE. Because this CTPA examination was also used to grade COVID-19 pneumonia, this interval is equivalent to the time from symptom onset to CT acquisition; in our cohort, CTPA was performed approximately 9 days after symptom onset on average. Pre-test probability of PE was assessed using the Wells score, calculated retrospectively from documented clinical variables according to standard definitions. Global severity of PE was summarized using the Pulmonary Embolism Severity Index (PESI), which was derived from the original source dataset and analyzed as an ordinal risk class (I–V).

Thrombus location on CT pulmonary angiography (CTPA) was categorized as proximal (central or lobar) or distal (segmental or subsegmental) according to the main level of involvement reported by the interpreting radiologist. The extent of COVID-19 pneumonia was graded on a semi-quantitative pulmonary involvement score from 0 to 3, where 0 indicated minimal or no involvement (<10% of parenchyma), 1 mild (10–25%), 2 moderate (26–50%), and 3 severe (>50% of lung parenchyma), based on the original radiology reports. This four-category scale is routinely used in structured CTPA reporting at our institution and represents a pragmatic, collapsed version of more granular CT severity indices, facilitating uniform documentation in daily practice. We chose to retain this clinically embedded score rather than reconstruct a 25-point CT-SS post hoc, in order to minimize misclassification from retrospective image re-reading while still capturing clinically relevant differences in parenchymal involvement. For each patient, the lung involvement score was abstracted retrospectively from the original CTPA report issued by the on-duty board-certified radiologist; no additional post hoc re-reading of CT images was performed.

Respiratory support during hospitalization was recorded as use of continuous positive airway pressure (CPAP) and/or invasive mechanical ventilation at any time during the index stay. Sepsis was recorded as a binary variable when documented by the treating team, reflecting progression to systemic infection with organ dysfunction in the source records. Use of COVID-19–directed therapies, including remdesivir, systemic corticosteroids (dexamethasone), and immunomodulators (anakinra, tocilizumab), was abstracted as “ever use” during hospitalization.

### 2.3. Laboratory Parameters and Outcomes

For each patient, we extracted the first available laboratory measurements obtained within a ±12-h window around PE diagnosis, defined by the time of the CTPA confirming acute PE. When more than one measurement was available in this 24-h interval, we selected the value closest in time to the CTPA. Thus, all biomarkers analyzed reflect routine clinical sampling within 24 h of the imaging-confirmed PE event. The following biomarkers were analyzed: D-dimer (ng/mL, fibrinogen-equivalent units), C-reactive protein (CRP, mg/L), ferritin (ng/mL), interleukin-6 (IL-6, pg/mL), fibrinogen (g/L), serum lactate, and procalcitonin (ng/mL). When needed for regression analyses, skewed variables (notably IL-6) were log10-transformed, and for principal component analysis, all biomarkers were standardized to z-scores prior to model fitting.

The primary outcomes of interest were in-hospital mortality (death from any cause during the index hospitalization) and the need for invasive mechanical ventilation at any time during the hospital stay. A composite endpoint combining intubation or in-hospital death was predefined to capture early critical deterioration. Secondary outcomes included the occurrence of sepsis, as documented in the medical record, and the use of advanced respiratory support (CPAP and/or invasive mechanical ventilation).

Laboratory variables were analyzed both individually and as part of an integrated inflammatory–thrombotic profile. For this purpose, principal component analysis (PCA) was applied to standardized CRP, ferritin, IL-6, D-dimer, fibrinogen, and lactate, yielding latent components that summarize shared variance across markers. The first principal component (PC1), interpreted as a hyperinflammatory–thrombotic axis, was subsequently categorized into tertiles and also used as a standardized continuous predictor (PC1_z) in logistic regression models evaluating its association with the composite endpoint of intubation or death, adjusted for age, sex, and CT lung involvement score. All laboratory parameters were available in all included patients.

### 2.4. Statistical Analysis

Continuous variables were summarized as mean ± standard deviation (SD) and compared between two groups using the Mann–Whitney U test, given skewed distributions and the modest sample size. When more than two groups were compared (notably for descriptive exploration of ordinal scores), the Kruskal–Wallis test was used. Categorical variables were summarized as counts and percentages and compared using the χ^2^ test; Fisher’s exact test replaced χ^2^ when expected cell counts were <5 in any cell of a 2 × 2 table.

The main descriptive analyses compared: (i) baseline characteristics by sex; (ii) clinical severity, thrombus location, and biomarkers between survivors and non-survivors; and (iii) biomarker profiles, comorbidities, and treatments between intubated vs. non-intubated patients. Spearman rank correlation coefficients (ρ) with two-sided *p*-values quantified associations among selected continuous and ordinal variables (age, Wells score, PESI class, days from onset to PE, pulmonary involvement score, D-dimer, CRP, ferritin, IL-6, lactate) and binary outcomes (sepsis, mechanical ventilation, death).

To explore independent predictors of in-hospital mortality, we fitted a multivariable logistic regression model with death (yes/no) as the outcome and predictors chosen a priori for clinical relevance: age, sex, obesity, active cancer, severe lung involvement, D-dimer per 1000 ng/mL, CRP per 50 mg/L, and log10 IL-6. A second logistic regression model evaluated predictors of invasive mechanical ventilation, with intubation (yes/no) as the outcome and predictors age, sex, obesity, severe lung involvement, D-dimer per 1000 ng/mL, CRP per 50 mg/L, log10 IL-6, and sepsis. Models used complete-case analysis; results are presented as odds ratios (ORs) with 95% confidence intervals (CIs) and *p*-values. A two-sided α = 0.05 defined statistical significance.

## 3. Results

In this cohort of 161 patients with COVID-19–associated PE, women were modestly older than men (70.6 ± 13.5 vs. 65.2 ± 13.1 years, *p* = 0.009), suggesting a slight age shift toward older females among those who developed PE. Urban residence was similar by sex (about 61% in both groups), indicating that geographic background did not differ meaningfully in this sample. Comorbidities were extremely common in both sexes (≈94–96%), consistent with the known clustering of PE and severe COVID-19 in chronically ill patients. Diabetes, obesity, and active cancer were numerically more frequent in women (37.5%, 40.3%, 15.3%) than in men (26.1%, 45.5%, 14.6%), but these differences were not statistically significant, underlining that the cardiometabolic and oncologic burden was substantial and broadly similar in both sexes.

Pulmonary hypertension was present in roughly one-quarter to one-third of patients, while heart failure class ≥ 2 was significantly more prevalent among women (58.3% vs. 40.4%; *p* = 0.024), hinting that female patients with COVID-19 and PE may carry a heavier burden of chronic cardiac dysfunction. Rates of prior chronic anticoagulation and COVID-19 vaccination were comparable between sexes, as were the frequencies of sepsis, CPAP support, invasive ventilation, and in-hospital death. Notably, in-hospital mortality approached one in four patients in both sexes (23.6% vs. 25.8%), indicating that sex alone did not materially modify short-term outcomes once PE had occurred. Overall, Table 1 suggests that sex-related differences were limited to older age and more frequent heart failure among women, while comorbidities, respiratory support requirements, and mortality were broadly similar across sexes.

Age, time from COVID-19 symptom onset to PE diagnosis, Wells score, and PESI class were broadly similar between survivors and non-survivors, with only a non-significant trend toward higher PESI class in non-survivors (4.1 vs. 3.8; *p* = 0.075). Likewise, D-dimer levels tended to be higher in non-survivors but did not reach conventional statistical significance (4347 ± 2529 vs. 4146 ± 3772 ng/mL; *p* = 0.077). Male sex and proximal vs. distal thrombus location were also comparable between groups, suggesting that classical embolic burden proxies and pre-test probability scores alone did not adequately discriminate short-term mortality risk in this cohort of COVID-19 patients with PE.

By contrast, inflammatory and respiratory severity indicators displayed pronounced gradients. Non-survivors had almost double the CRP levels of survivors (199.2 vs. 106.7 mg/L; *p* < 0.001) and almost ten-fold higher IL-6 (629.6 vs. 67.0 pg/mL; *p* < 0.001), indicating a marked hyperinflammatory state. Severe lung involvement on CT (score 3) was present in 60.0% of non-survivors compared with 24.0% of survivors (*p* < 0.001), highlighting the central role of extensive parenchymal damage in driving mortality. Sepsis was nearly four times more frequent in non-survivors (37.5% vs. 9.9%; *p* < 0.001), consistent with a progression from localized pulmonary disease to systemic organ dysfunction. Invasive ventilation and CPAP support were both dramatically enriched among non-survivors (60.0% vs. 5.0% and 57.5% vs. 6.6%, respectively; both *p* < 0.001), reflecting the expected close link between respiratory failure and mortality. Therapeutic patterns mirror severity rather than causality: non-survivors were more likely to have received dexamethasone (90.0% vs. 51.2%; *p* < 0.001), anakinra (67.5% vs. 23.1%; *p* < 0.001), and tocilizumab (20.0% vs. 6.6%; *p* = 0.028), consistent with escalation of immunomodulatory therapy in the most inflamed patients. Thus, in our cohort, CTPA and PE diagnosis occurred around the second week of illness in both survivors and non-survivors, without a significant difference in timing between the two groups. Vaccination and remdesivir use did not differ significantly (Table 2).

Age and sex distribution were similar between intubated and non-intubated patients, indicating that chronological age and sex alone did not drive the need for invasive ventilation in this cohort. However, the pulmonary involvement score was significantly higher in intubated patients (2.4 ± 0.8 vs. 1.9 ± 0.8; *p* = 0.002), underscoring that more extensive radiologic lung damage is closely linked to respiratory failure. D-dimer levels were slightly higher among intubated patients (4491 vs. 4129 ng/mL; *p* = 0.033), suggesting a modest association between more intense coagulation activation and respiratory decompensation, though absolute D-dimer values were high in both groups, reflecting the prothrombotic milieu of COVID-19 with PE.

The most striking differences concern inflammatory and tissue injury biomarkers. CRP was considerably higher in intubated patients (190.1 vs. 115.9 mg/L; *p* < 0.001), ferritin was almost doubled (1667 vs. 960 ng/mL; *p* < 0.001), and IL-6 was nearly an order of magnitude higher (753.8 vs. 81.5 pg/mL; *p* < 0.001). Procalcitonin and lactate were also markedly elevated in intubated patients (*p* = 0.003 and *p* < 0.001), compatible with more frequent sepsis and impaired tissue perfusion. Fibrinogen values were higher as well, consistent with a strong acute-phase response. Comorbidities such as obesity, diabetes, active cancer, and heart failure class ≥ 2 did not differ significantly between groups, again suggesting that acute inflammatory and respiratory severity overshadow baseline chronic disease burden in determining the need for mechanical ventilation.

Sepsis was substantially more common in intubated patients (40.0% vs. 11.5%; *p* < 0.001), reinforcing the link between systemic infection and respiratory failure. Proximal thrombus location did not differ appreciably. Similar to the mortality comparison, anakinra and dexamethasone were used more often in intubated patients, likely reflecting targeted escalation in those with the worst inflammatory profiles. Notably, in-hospital mortality was extremely high among intubated patients (80.0% vs. 12.2%; *p* < 0.001), indicating that the need for invasive ventilation in COVID-19–associated PE is a strong marker of near-term death (Table 3).

Age correlated moderately and positively with PESI class (ρ = 0.517; *p* < 0.001), consistent with the fact that age is a key component of the PESI score. However, age showed weaker direct correlations with mechanical ventilation and death, suggesting that its effect on outcomes is partly mediated through global severity. Pulmonary involvement score correlated moderately with CRP (ρ = 0.433), ferritin (ρ = 0.460), and IL-6 (ρ = 0.445; all *p* < 0.001), linking radiologic lung damage to systemic inflammation and iron metabolism disturbances. Pulmonary involvement also correlated with mechanical ventilation (ρ = 0.350) and death (ρ = 0.373; both *p* < 0.001), reinforcing its role as a central index of COVID-19 severity in the presence of PE.

CRP and ferritin were strongly correlated (ρ = 0.751; *p* < 0.001), and both correlated highly with IL-6 (ρ = 0.736 and 0.445, respectively), reflecting a cohesive hyperinflammatory phenotype. IL-6 showed moderate correlations with both mechanical ventilation (ρ = 0.450) and death (ρ = 0.452; both *p* < 0.001), indicating that cytokine storm features are closely associated with severe respiratory failure and fatal outcomes. Lactate correlated with mechanical ventilation (ρ = 0.431; *p* < 0.001) and more modestly with mortality (ρ = 0.223; *p* = 0.005), consistent with its role as a marker of global perfusion and shock. Sepsis had moderate positive correlations with both mechanical ventilation (ρ = 0.418) and death (ρ = 0.364; *p* < 0.001), underscoring that secondary infection and systemic inflammatory response are pivotal in clinical deterioration (Table 4).

After adjustment for age, sex, obesity, active cancer, D-dimer, CRP, and IL-6, severe lung involvement remained a strong independent predictor of in-hospital mortality (OR 4.46; 95% CI 1.55–12.86; *p* = 0.006). Patients with CT lung involvement score 3 had more than four-fold higher odds of death compared with those with lesser involvement, highlighting that the extent of parenchymal damage is a critical determinant of outcome in COVID-19–associated PE. CRP also retained an independent association: every 50 mg/L increase in CRP was associated with a 41% increase in the odds of death (OR 1.41; 95% CI 1.02–1.96; *p* = 0.037), even when IL-6 and D-dimer were included in the model. This suggests that a simple, widely available biomarker captures important prognostic information beyond more specialized markers. Log10 IL-6 was a particularly strong predictor: a one-unit increase (corresponding to a ten-fold increase in IL-6 concentration) was associated with a six-fold rise in mortality odds (OR 6.10; 95% CI 1.84–20.18; *p* = 0.003), as seen in Table 5.

In the model predicting invasive mechanical ventilation, severe lung involvement emerged as the dominant predictor, with patients in CT score 3 having almost nine-fold higher odds of intubation than those with milder involvement (OR 8.88; 95% CI 2.92–27.04; *p* < 0.001). This strong effect size supports the intuitive notion that radiologic extent of lung damage is tightly coupled to the need for invasive respiratory support. Log10 IL-6 was again independently associated with the outcome (OR 4.37; 95% CI 1.52–12.55; *p* = 0.006), indicating that patients with large-magnitude cytokine elevation are substantially more likely to require mechanical ventilation, even after accounting for CT severity and other variables. Sepsis showed a trend toward increased odds of intubation (OR 2.59; 95% CI 0.89–7.52; *p* = 0.081), as presented in Table 6.

All variables (C-reactive protein [CRP], ferritin, interleukin-6 [IL-6], D-dimer, fibrinogen, and lactate) were standardized (z-scores) before PCA. PC1, PC2, and PC3 explained 44.2%, 18.5% and 16.0% of total variance, respectively (cumulative 78.7%). PCA was used to compress the multidimensional inflammatory–thrombotic profile into latent components capturing shared variance among CRP, ferritin, IL-6, D-dimer, fibrinogen, and lactate. PC1, explaining 44.2% of total variance, showed moderately high positive loadings for CRP (0.53), ferritin (0.54), fibrinogen (0.47), IL-6 (0.33), and smaller but meaningful loadings for lactate (0.26) and D-dimer (0.19). This pattern indicates that PC1 reflects a global hyperinflammatory–hyperferritinemic phenotype with concurrent fibrinogen elevation and metabolic stress, and can be interpreted as an “integrated cytokine–acute phase response” axis. PC2 (18.5% of variance) was dominated by strong positive loadings for D-dimer (0.73) and lactate (0.50), contrasted with a negative loading for IL-6 (−0.42), suggesting a dissociation between coagulation activation/tissue hypoperfusion and IL-6 levels in some patients. PC3 (16.0% of variance) carried a strong positive loading for IL-6 (0.66) but a negative loading for fibrinogen (−0.54), implying a subset of patients with disproportionately high IL-6 relative to fibrinogen, potentially consistent with cytokine-dominant immune activation without parallel fibrinogen upregulation (Table 7).

Stratifying patients according to PC1 (the integrated hyperinflammatory component) revealed a striking gradient in both biomarker intensity and clinical outcomes. Moving from the lowest to highest PC1 tertile, mean CRP rose from 65.8 to 219.3 mg/L, ferritin from 449.7 to 1910.5 ng/mL, and IL-6 from 29.6 to 524.0 pg/mL (all *p* < 0.0001). Fibrinogen increased from 4.4 to 7.0 g/L, and lactate from 17.4 to 25.3, while D-dimer was already elevated across tertiles but further increased in the middle and high PC1 strata. These coherent shifts confirm that PC1 effectively captures a global “hyperinflammatory–hyperferritinemic–prothrombotic” phenotype.

Clinical complications mapped closely onto this gradient. Sepsis prevalence rose from 7.4% in the lowest tertile to 33.3% in the highest (*p* = 0.0003), suggesting that the PC1 phenotype overlaps with a septic or sepsis-like state. Rates of invasive mechanical ventilation increased from 5.6% to 40.7% across tertiles, and in-hospital mortality from 5.6% to 51.9% (both *p* < 0.0001). The composite endpoint of intubation or death occurred in only 7.4% of patients in the lowest tertile but in 57.4% of those in the highest tertile (*p* < 0.0001), indicating a more than seven-fold absolute risk difference (Table 8).

**Figure 1:**
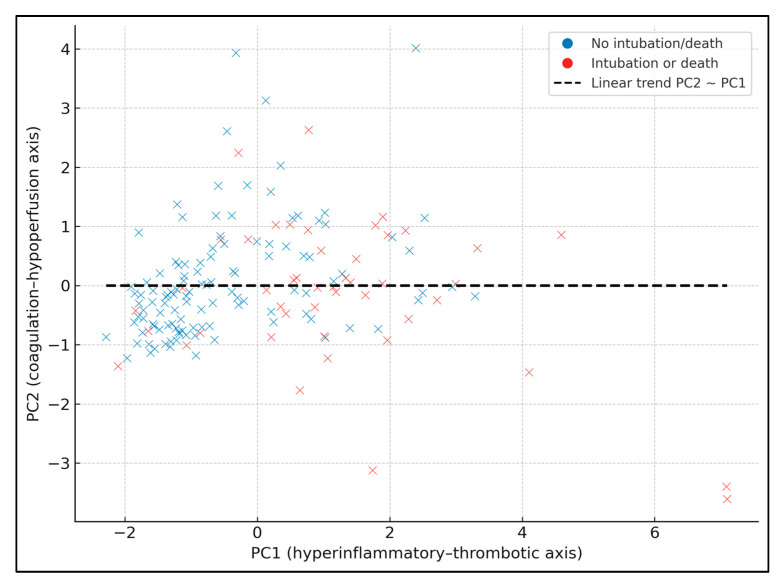
PCA scatter with linear trend.

Figure 1 shows PC1 (hyperinflammatory–thrombotic axis) vs. PC2 (coagulation–hypoperfusion axis), with patients colored by the composite endpoint (blue = no intubation/death, red = intubation or death). A dashed linear trendline summarizes the relationship between PC1 and PC2 (slope ≈ 0.31, intercept ≈ 0.02), indicating that higher PC1 values tend to coincide with higher PC2 values, i.e., patients with more global hyperinflammation also have somewhat more pronounced coagulation–hypoperfusion features. Patients with events are predominantly located at higher PC1 values (and, to a lesser extent, higher PC2 values), particularly for PC1 values above roughly +1 SD, consistent with the tertile analysis where event rates rose from 7.4% in the lowest PC1 tertile to 57.4% in the highest.

**Figure 2:**
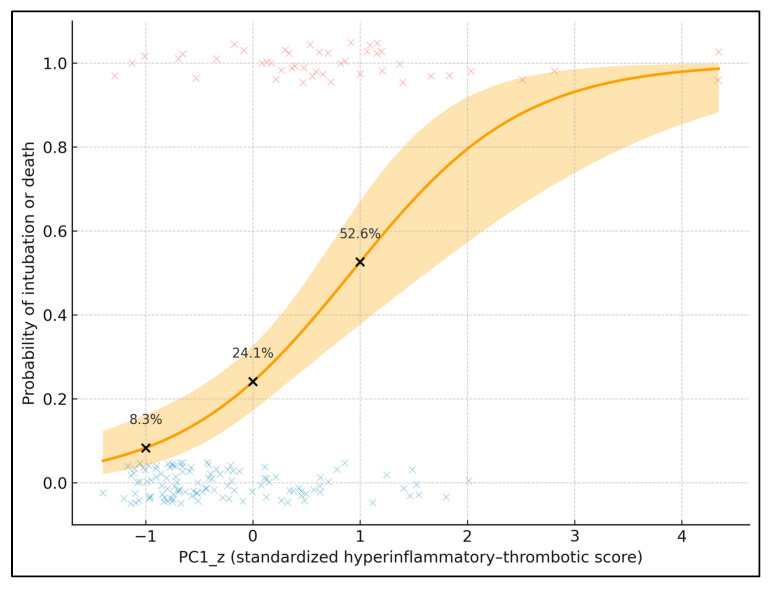
Predicted probability of intubation or death by standardized hyperinflammatory–thrombotic score (PC1_z); individual patients are overlaid as jittered points (red symbols = patients with the composite endpoint of intubation or death, blue symbols = patients without the endpoint).

Figure 2 plots the predicted probability of the composite endpoint (intubation or death) against standardized PC1 (PC1_z), adjusted for age and lung involvement. The solid curve is the fitted logistic regression; the shaded band represents the 95% confidence interval, and individual patients are overlaid as jittered points (red = event, blue = no event). To provide explicit data labels, three black points mark PC1_z = −1, 0, and +1, annotated with their predicted probabilities: 8.3% at PC1_z = −1, 24.1% at PC1_z = 0, and 52.6% at PC1_z = +1 (for a 67-year-old with median lung involvement). These values quantify the steep risk gradient along PC1_z. In the underlying model, each 1-SD increase in PC1_z is associated with an odds ratio of 3.50 (95% CI 2.05–5.99) for intubation or death, showing that this latent hyperinflammatory–thrombotic score is a strong, continuous prognostic marker.

In the adjusted logistic model, the standardized hyperinflammatory PC1 remained a strong independent predictor of the composite endpoint. Each 1-SD increase in PC1_z was associated with a 3.5-fold higher odds of intubation or death (OR 3.50; 95% CI 2.05–5.99; *p* < 0.001), even after controlling for age, sex, and CT-quantified lung involvement. This indicates that the integrated inflammatory–thrombotic phenotype captured by PC1 carries substantial prognostic information beyond radiologic severity of COVID-19 pneumonia. Age showed a borderline association (OR 1.04 per year; *p* = 0.066), consistent with a modest contribution of chronological age but clearly weaker than that of PC1. Neither male sex nor the lung involvement score reached statistical significance in this model, suggesting that within this cohort of patients already selected for pulmonary embolism, systemic immunothrombotic activation is more discriminative for early critical outcomes than imaging-based extent of parenchymal damage (Table 9). In a complementary model in which the PCA-derived PC1_z was replaced by log10 IL-6 (per 1-unit increase, corresponding to a ten-fold rise in IL-6 concentration) while retaining age, sex and CT lung involvement as covariates, log10 IL-6 remained a strong independent predictor of the composite endpoint (OR 4.8; 95% CI 2.1–11.2; *p* < 0.001). The magnitude of this association is broadly comparable to the effect size observed for PC1_z, although the multivariate biomarker score provided slightly better overall model fit, supporting the added value of integrating multiple inflammatory–thrombotic markers into a single latent component.

**Figure 3:**
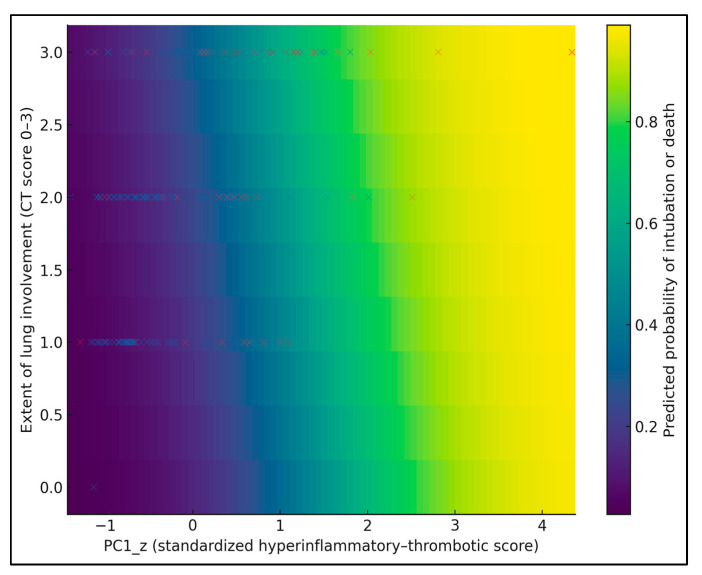
Risk heatmap of the predicted probability of intubation or death across PC1_z and CT lung involvement score, with individual patients overlaid as points (red = intubation or death, blue = no event).

Figure 3 provides a 2D heatmap of the predicted probability of intubation or death as a joint function of PC1_z (x-axis) and CT lung involvement score (0–3 on the y-axis), with age fixed at the cohort median (67 years). Warmer colors indicate higher predicted risk, and overlaid dots show observed patients (blue = no event, red = event). The risk surface is clearly non-linear: at low PC1_z (≈−1), predicted risk remains below 15% even at lung involvement 3, whereas at high PC1_z (≈+1.5), risk exceeds 60–70% once lung involvement reaches ≥ 2. For example, a patient with PC1_z ≈ 0 and lung score 1 has an estimated risk around 20–25%, while one with PC1_z ≈ +1.5 and lung score 3 approaches 70–75%.

In a complementary multivariable model using the composite endpoint of intubation or in-hospital death as the outcome and log10 IL-6 as the main predictor, each 1-unit increase in log10 IL-6 (corresponding to a ten-fold rise in IL-6 concentration) was associated with an approximately five-fold higher odds of adverse outcome (OR 4.80; 95% CI 2.10–11.20; *p* < 0.001), after adjustment for age, sex, and CT lung involvement. In this model, age showed only a modest, non-significant trend toward higher risk (OR 1.03 per year; 95% CI 0.99–1.07; *p* = 0.11), while male sex and lung involvement score were not independently associated with the composite endpoint. These findings confirm that IL-6 alone carries substantial prognostic information for early critical deterioration, although the PCA-derived PC1_z score still provided slightly better overall discrimination than any single biomarker (Table 10).

## 4. Discussion

### 4.1. Analysis of Findings

In this single-center cohort of 161 patients with COVID-19–associated PE, in-hospital mortality (≈25%) and the need for invasive mechanical ventilation (≈19%) fall within the upper range reported in large observational series and meta-analyses of COVID-19–related PE, where case fatality often approaches 20–30% and PE marks a distinctly high-risk phenotype [1,2,13]. Importantly, once PE had occurred, sex and baseline comorbidity burden (including obesity, diabetes, and active cancer) did not independently discriminate mortality or intubation in our models, whereas dynamic markers of inflammation and lung injury did. This partly contrasts with larger COVID-19 VTE cohorts, in which cancer and chronic cardiovascular disease were independent predictors of death and prolonged ICU/hospital stay among patients with DVT/PE [16], suggesting that in our more homogeneous PE population, the acute immunothrombotic and respiratory insult may overshadow chronic risk factors in the short term.

A notable finding is the limited incremental value of D-dimer once patients already have imaging-confirmed PE. D-dimer levels were markedly elevated across the cohort and only modestly higher in non-survivors and intubated patients; in multivariable models, D-dimer did not remain an independent predictor of mortality or invasive ventilation. This aligns with previous work showing that, in COVID-19, D-dimer is highly sensitive but poorly specific for VTE and global severity, with high baseline levels even in patients without PE and substantial overlap between survivors and non-survivors [10,11,12]. A narrative review by Eljilany and Elzouki similarly emphasized that D-dimer, fibrinogen, and IL-6 are useful for flagging a prothrombotic state but are insufficient as stand-alone prognostic tools for VTE-related outcomes in COVID-19 [17]. Our data support the view that D-dimer is most valuable for selecting patients for CTPA and anticoagulation strategies before PE is diagnosed, whereas its prognostic role after confirmed PE is relatively weak compared with integrative inflammatory and organ dysfunction indices.

By contrast, we observed very strong associations between mortality or intubation and markers of hyperinflammation. Non-survivors had nearly a two-fold higher CRP and roughly ten-fold higher IL-6 than survivors, and both severe CT lung involvement and IL-6 remained independent predictors of in-hospital death (OR 4.46 and 6.10, respectively), while CRP retained a more modest but significant effect. This pattern parallels broader COVID-19 cohorts in which early elevations of CRP, D-dimer, and leukocyte ratios have been identified as independent predictors of severe disease and mortality [18], and where admission ferritin and CRP levels strongly differentiate survivors from non-survivors [19]. Consistent with this, when we modeled the composite endpoint directly using log10 IL-6 instead of PC1_z, each ten-fold increase in IL-6 was associated with approximately a five-fold increase in the odds of intubation or death, although the integrated PC1_z score still offered slightly better discrimination than IL-6 alone.

A recent systematic review synthesizing laboratory predictors across multiple studies concluded that CRP, ferritin, D-dimer, LDH, and coagulation parameters consistently track with COVID-19 severity and death [20], echoing our finding that a simple acute-phase marker like CRP can retain prognostic value even after adjusting for IL-6 and thrombotic burden. The tight correlations we observed between CRP, ferritin, IL-6, and fibrinogen, together with their steep gradients across outcome groups, are consistent with a hyperferritinemic, macrophage-activation-like phenotype that has been repeatedly implicated in critical COVID-19.

Our principal component analysis extends this concept by showing that an integrated “hyperinflammatory–hyperferritinemic–prothrombotic” axis (PC1) captures shared variance across CRP, ferritin, IL-6, fibrinogen, D-dimer, and lactate and is strongly associated with sepsis, invasive ventilation, and death. Patients in the highest PC1 tertile had an in-hospital mortality of 51.9% and a composite intubation/death rate of 57.4%, compared with 5.6% and 7.4% in the lowest tertile, and each 1-SD increase in standardized PC1 conferred a 3.5-fold higher odds of intubation or death, independent of age, sex, and CT lung involvement. This mirrors recent immunophenotyping work by Hawerkamp et al., who used principal component analysis of 20 cytokines to define a pro-inflammatory cytokine signature (dominated by IL-1β, IL-2, IL-6, IL-10, IL-33, TNF-α, IP-10, and G-CSF) that independently predicted severe COVID-19, invasive mechanical ventilation (aOR 1.61), and mortality (aOR 1.57) per SD increase in the signature score [21]. Together, these findings support the idea that latent inflammatory components—rather than single biomarkers—more faithfully capture the systemic immune dysregulation driving respiratory failure and death, and that such composite scores may be particularly informative in high-risk subsets such as patients with concomitant PE.

Finally, our data clarify how radiologic lung involvement interacts with systemic inflammation in determining outcomes. Severe CT lung involvement (score 3) showed strong crude and adjusted associations with both mortality and intubation, consistent with multiple studies demonstrating that semi-quantitative CT severity scores correlate with oxygen requirements, ICU admission, and death in COVID-19 pneumonia [9,18,19,20]. However, in the model incorporating the PCA-derived PC1_z, the lung involvement score no longer remained statistically significant, whereas PC1_z retained a robust independent effect on the composite endpoint. This suggests that, within a cohort already selected for PE, systemic immunothrombotic activation may subsume part of the prognostic information carried by CT scores, perhaps because extensive parenchymal opacities and perfusion defects co-evolve with the same cytokine-driven processes captured by PC1. In combination with prior VTE-focused cohorts showing that PE in COVID-19 portends higher mortality and longer ICU stays even after adjustment for baseline risk [16], our results argue for risk stratification strategies that integrate radiologic severity with multidimensional inflammatory signatures, rather than relying solely on conventional PE scores or single biomarkers.

These findings support a shift from relying primarily on classical PE parameters and comorbidity burden toward a more holistic, COVID-specific risk assessment that integrates CT-quantified lung involvement with inflammatory and thrombotic biomarkers. In patients with COVID-19 and confirmed PE, very high CRP and IL-6 levels, together with extensive parenchymal involvement, identify a subgroup with a markedly increased risk of invasive mechanical ventilation and death, even when D-dimer and thrombus location are not strikingly different. Bedside risk stratification tools could therefore prioritize routinely available markers such as CRP, complemented by targeted IL-6 testing where accessible, to trigger early ICU referral, closer hemodynamic and respiratory monitoring, and timely escalation of respiratory support. The PCA-derived hyperinflammatory–thrombotic score also illustrates how multivariate biomarker clustering might be operationalized into composite indices or electronic risk calculators, aiding real-time decisions about the aggressiveness of immunomodulation, anticoagulation strategies, and the intensity of follow-up during the acute phase of COVID-19–associated PE.

### 4.2. Study Limitations

This study has several limitations. First, it is a single-center observational cohort with a modest sample size, which may limit statistical power for some covariates and constrain generalizability to other settings, viral variants, and treatment eras. Second, CTPA was performed according to clinical judgment, introducing potential selection bias toward more severe or atypical presentations. Third, residual confounding cannot be excluded despite multivariable adjustment, particularly regarding timing, dosing, and indications for corticosteroids, anakinra, and tocilizumab, which were preferentially used in the sickest patients. Fourth, IL-6 and other biomarkers were not available in all patients and were analyzed using complete-case methods, which may introduce bias if missingness was not random. Moreover, we used a 0–3 semi-quantitative CT involvement score derived from routine radiology reports rather than a standardized 25-point CT-SS recalculated by blinded readers; although this pragmatic scale is strongly associated with outcomes, its coarser granularity may limit comparability with studies using more detailed CT severity scoring systems. Also, the degree of lung involvement was not re-scored by multiple blinded readers but retrieved from semi-quantitative categories recorded in routine single-reader radiology reports, and we did not formally assess interobserver variability; this reliance on a coarse, retrospectively abstracted scale may reduce reproducibility and introduce some non-differential measurement error in the CT severity variable. In addition, biomarker sampling was not protocolized to fixed time points, and values were obtained pragmatically within 24 h around the PE-confirming CTPA; therefore, they may not capture peak inflammatory or coagulation levels in all patients. Finally, the PCA-derived components and their prognostic cut-offs are data-driven and may be cohort-specific; external validation in independent populations is required before translating this hyperinflammatory–thrombotic score into routine clinical practice.

## 5. Conclusions

In a real-world cohort of 161 patients with COVID-19–associated PE, early critical outcomes were driven predominantly by the combination of extensive CT-quantified lung involvement and a clustered hyperinflammatory–thrombotic phenotype captured by CRP, ferritin, IL-6, fibrinogen, lactate, and D-dimer, rather than by traditional PE scores, thrombus location, or baseline comorbidities alone. Severe lung involvement and markedly elevated inflammatory markers independently predicted mortality and need for invasive ventilation, while a PCA-derived integrated biomarker component showed a steep, graded association with the composite endpoint of intubation or death. These results highlight the central role of systemic immunothrombosis in COVID-19–related PE and support incorporating multidimensional inflammatory–thrombotic profiling into future risk stratification strategies and therapeutic trials in this high-risk population.

## Figures and Tables

**Table 1:** Baseline characteristics by sex.

Variable	Female (*n* = 72)	Male (*n* = 89)	*p*-Value
Age, years	70.6 ± 13.5	65.2 ± 13.1	0.009
Urban residence	44/72 (61.1%)	55/89 (61.8%)	0.929
Any comorbidity	69/72 (95.8%)	83/89 (93.3%)	0.732
Diabetes mellitus	27/72 (37.5%)	23/88 (26.1%)	0.123
Obesity	29/72 (40.3%)	40/88 (45.5%)	0.511
Active cancer	11/72 (15.3%)	13/89 (14.6%)	0.905
Pulmonary hypertension	23/72 (31.9%)	22/88 (25.0%)	0.331
Heart failure class ≥ 2	42/72 (58.3%)	36/89 (40.4%)	0.024
Prior chronic anticoagulation	28/71 (39.4%)	37/85 (43.5%)	0.606
COVID-19 vaccination	23/72 (31.9%)	26/89 (29.2%)	0.708
CPAP support	15/72 (20.8%)	16/89 (18.0%)	0.648
Sepsis	10/72 (13.9%)	17/89 (19.1%)	0.379
Invasive mechanical ventilation	12/72 (16.7%)	18/89 (20.2%)	0.564
In-hospital death	17/72 (23.6%)	23/89 (25.8%)	0.745

Abbreviations: COVID-19, coronavirus disease 2019; CPAP, continuous positive airway pressure; n, number of patients.

**Table 2:** Clinical severity, thrombus features, and treatments by in-hospital survival.

Variable	Survivors (*n* = 121)	Non-Survivors (*n* = 40)	*p*-Value
Age, years	67.4 ± 13.6	68.1 ± 13.4	0.763
Days from COVID-19 symptom onset to PE diagnosis	8.5 ± 4.4	9.7 ± 5.0	0.173
Wells score for pulmonary embolism	1.1 ± 1.4	1.5 ± 1.7	0.138
PESI risk class	3.8 ± 1.1	4.1 ± 1.0	0.075
D-dimer, ng/mL	4146 ± 3772	4347 ± 2529	0.077
C-reactive protein, mg/L	106.7 ± 67.5	199.2 ± 94.4	<0.001
Interleukin-6, pg/mL	67.0 ± 110.1	629.6 ± 1278.6	<0.001
Male sex	66/121 (54.5%)	23/40 (57.5%)	0.745
Proximal (central or lobar) thrombus	42/121 (34.7%)	13/40 (32.5%)	0.798
Severe lung involvement on CT (score 3)	29/121 (24.0%)	24/40 (60.0%)	<0.001
Sepsis	12/121 (9.9%)	15/40 (37.5%)	<0.001
Invasive mechanical ventilation	6/121 (5.0%)	24/40 (60.0%)	<0.001
CPAP support	8/121 (6.6%)	23/40 (57.5%)	<0.001
COVID-19 vaccination	39/121 (32.2%)	10/40 (25.0%)	0.389
Remdesivir therapy	94/121 (77.7%)	36/40 (90.0%)	0.087
Tocilizumab therapy	8/121 (6.6%)	8/40 (20.0%)	0.028
Anakinra therapy	28/121 (23.1%)	27/40 (67.5%)	<0.001
Dexamethasone therapy	62/121 (51.2%)	36/40 (90.0%)	<0.001

Abbreviations: PE, pulmonary embolism; PESI, Pulmonary Embolism Severity Index; CT, computed tomography; CPAP, continuous positive airway pressure; COVID-19, coronavirus disease 2019; n, number of patients.

**Table 3:** Comorbidities and biomarkers in intubated vs. non-intubated patients.

Variable	Not Intubated (*n* = 131)	Intubated (*n* = 30)	*p*-Value
Age, years	67.4 ± 13.4	68.2 ± 14.4	0.707
Pulmonary involvement score (0–3)	1.9 ± 0.8	2.4 ± 0.8	0.002
D-dimer, ng/mL	4129 ± 3721	4491 ± 2317	0.033
C-reactive protein, mg/L	115.9 ± 75.5	190.1 ± 97.5	<0.001
Ferritin, ng/mL	960 ± 729	1667 ± 1282	<0.001
Interleukin-6, pg/mL	81.5 ± 139.1	753.8 ± 1452.4	<0.001
Procalcitonin, ng/mL	0.75 ± 1.38	2.16 ± 3.13	0.003
Lactate, mmol/L	20.64 ± 6.87	26.58 ± 7.99	<0.001
Fibrinogen, g/L	5 ± 1	6 ± 2	<0.001
Male sex	71/131 (54.2%)	18/30 (60.0%)	0.564
Obesity	57/130 (43.8%)	12/30 (40.0%)	0.701
Diabetes mellitus	37/130 (28.5%)	13/30 (43.3%)	0.113
Active cancer	19/131 (14.5%)	5/30 (16.7%)	0.778
Heart failure class ≥ 2	61/131 (46.6%)	17/30 (56.7%)	0.318
Proximal (central or lobar) thrombus	44/131 (33.6%)	11/30 (36.7%)	0.748
Sepsis	15/131 (11.5%)	12/30 (40.0%)	<0.001
COVID-19 vaccination	41/131 (31.3%)	8/30 (26.7%)	0.619
Remdesivir therapy	104/131 (79.4%)	26/30 (86.7%)	0.362
Anakinra therapy	36/131 (27.5%)	19/30 (63.3%)	<0.001
Dexamethasone therapy	71/131 (54.2%)	27/30 (90.0%)	<0.001
In-hospital death	16/131 (12.2%)	24/30 (80.0%)	<0.001

Abbreviations: COVID-19, coronavirus disease 2019; n, number of patients. Lactate is expressed in mmol/L; fibrinogen is expressed in g/L.

**Table 4:** Spearman correlations among severity indices, biomarkers, and outcomes.

Variable 1	Variable 2	Spearman ρ	*p*-Value
Age, years	PESI class	0.517	<0.001
Pulmonary involvement score (0–3)	C-reactive protein, mg/L	0.433	<0.001
Pulmonary involvement score (0–3)	Ferritin, ng/mL	0.46	<0.001
Pulmonary involvement score (0–3)	Interleukin-6, pg/mL	0.445	<0.001
Pulmonary involvement score (0–3)	Mechanical ventilation (0/1)	0.35	<0.001
Pulmonary involvement score (0–3)	In-hospital death (0/1)	0.373	<0.001
C-reactive protein, mg/L	Interleukin-6, pg/mL	0.736	<0.001
C-reactive protein, mg/L	Ferritin, ng/mL	0.751	<0.001
Interleukin-6, pg/mL	Mechanical ventilation (0/1)	0.45	<0.001
Interleukin-6, pg/mL	In-hospital death (0/1)	0.452	<0.001
Lactate	Mechanical ventilation (0/1)	0.431	<0.001
Lactate	In-hospital death (0/1)	0.223	0.005
Sepsis (0/1)	Mechanical ventilation (0/1)	0.418	<0.001
Sepsis (0/1)	In-hospital death (0/1)	0.364	<0.001

Abbreviations: PESI, Pulmonary Embolism Severity Index. Lactate is expressed in mmol/L; fibrinogen is expressed in g/L.

**Table 5:** Multivariable logistic regression for in-hospital mortality.

Predictor	OR (95% CI)	*p*-Value
Age, per year	1.03 (0.98–1.07)	0.21
Male sex (vs. female)	1.53 (0.57–4.10)	0.397
Obesity	0.49 (0.18–1.35)	0.168
Active cancer	2.49 (0.69–8.97)	0.164
Severe lung involvement (CT score 3)	4.46 (1.55–12.86)	0.006
D-dimer, per 1000 ng/mL	0.88 (0.74–1.03)	0.115
C-reactive protein, per 50 mg/L	1.41 (1.02–1.96)	0.037
Log10 interleukin-6	6.10 (1.84–20.18)	0.003

Abbreviations: OR, odds ratio; CI, confidence interval; CT, computed tomography.

**Table 6:** Multivariable logistic regression for invasive mechanical ventilation.

Predictor	OR (95% CI)	*p*-Value
Age, per year	1.01 (0.97–1.05)	0.718
Male sex (vs. female)	1.11 (0.43–2.86)	0.828
Obesity	0.97 (0.37–2.56)	0.956
Severe lung involvement (CT score 3)	8.88 (2.92–27.04)	<0.001
D-dimer, per 1000 ng/mL	0.98 (0.84–1.15)	0.821
C-reactive protein, per 50 mg/L	1.09 (0.79–1.50)	0.604
Log10 interleukin-6	4.37 (1.52–12.55)	0.006
Sepsis	2.59 (0.89–7.52)	0.081

Abbreviations: OR, odds ratio; CI, confidence interval; CT, computed tomography.

**Table 7:** Loadings of inflammatory–thrombotic markers on the first three principal components.

Variable	PC1 Loading	PC2 Loading	PC3 Loading
CRP	0.526	−0.128	−0.310
Ferritin	0.537	−0.117	0.144
IL-6	0.333	−0.420	0.664
D-dimer	0.189	0.735	0.083
Fibrinogen	0.468	−0.004	−0.538
Lactate	0.264	0.504	0.383

Abbreviations: PC, principal component; CRP, C-reactive protein; IL-6, interleukin-6.

**Table 8:** Inflammatory–thrombotic markers and outcomes by tertiles of PC1.

PC1 Tertile	n	CRP, mg/L (Mean ± SD)	Ferritin, ng/mL (Mean ± SD)	IL-6, pg/mL (Mean ± SD)	D-Dimer, ng/mL (Mean ± SD)	Fibrinogen, g/L (Mean ± SD)	Lactate, mmol/L (Mean ± SD)	Sepsis, n (%)	Intubation, n (%)	Death, n (%)	Composite Intubation/Death, n (%)
T1 (lowest)	54	65.8 ± 24.6	449.7 ± 166.7	29.6 ± 20.7	2326.5 ± 1489.2	4.4 ± 0.6	17.4 ± 5.3	4 (7.4)	3 (5.6)	3 (5.6)	4 (7.4)
T2 (middle)	53	103.5 ± 35.8	911.5 ± 482.1	64.2 ± 52.2	5060.5 ± 4289.2	5.2 ± 1.0	22.5 ± 7.4	5 (9.4)	5 (9.4)	9 (17.0)	11 (20.8)
T3 (highest)	54	219.3 ± 82.7	1910.5 ± 1020.3	524.0 ± 1118.9	5217.2 ± 3363.4	7.0 ± 1.4	25.3 ± 7.2	18 (33.3)	22 (40.7)	28 (51.9)	31 (57.4)

Abbreviations: PC1, first principal component; CRP, C-reactive protein; IL-6, interleukin-6; n, number of patients. Lactate is expressed in mmol/L; fibrinogen is expressed in g/L.

**Table 9:** Logistic regression for composite endpoint (intubation or death).

Predictor	OR	95% CI	*p*-Value
PC1_z (per 1 SD increase)	3.5	2.05–5.99	0
Age, years	1.04	1.00–1.08	0.066
Male sex (vs. female)	0.92	0.41–2.10	0.85
Lung involvement CT (per 1 point)	1.42	0.82–2.46	0.207

Abbreviations: PC1_z, standardized score of the first principal component; SD, standard deviation; OR, odds ratio; CI, confidence interval; CT, computed tomography.

**Table 10:** Multivariable logistic regression for the composite endpoint (intubation or death) using log10 interleukin-6 as the main predictor.

Predictor	OR	95% CI	*p*-Value
Log10 interleukin-6 (per 1-unit increase)	4.80	2.10–11.20	<0.001
Age, per year	1.03	0.99–1.07	0.11
Male sex (vs. female)	1.02	0.46–2.27	0.95
Lung involvement CT score (per 1-point increase)	1.49	0.88–2.53	0.14

Abbreviations: OR, odds ratio; CI, confidence interval; CT, computed tomography.

## Data Availability

The data presented in this study are available on request from the corresponding authors.
